# Long-term acceptability, durability and bio-efficacy of ZeroVector^®^ durable lining for vector control in Papua New Guinea

**DOI:** 10.1186/s12936-017-1742-y

**Published:** 2017-02-28

**Authors:** Joseph J. Kuadima, Lincoln Timinao, Laura Naidi, Anthony Tandrapah, Manuel W. Hetzel, Cyrille Czeher, Justin Pulford

**Affiliations:** 1 Papua New Guinea Institute of Medical Research (PNGIMR), Goroka, EHP 441 Papua New Guinea; 2 Swiss Tropical and Public Health Institute, PO Box 4002, Basel, Switzerland; 3 University of Basel, Petersplatz 1, 4003 Basel, Switzerland; 4 Liverpool School of Tropical Medicine, Liverpool, Pembroke Place, Liverpool, l35QA UK

**Keywords:** Vector control, Durable lining, Papua New Guinea

## Abstract

**Background:**

This study examined the acceptability, durability and bio-efficacy of pyrethroid-impregnated durable lining (DL) over a three-year period post-installation in residential homes across Papua New Guinea (PNG).

**Methods:**

ZeroVector^®^ ITPS had previously been installed in 40 homes across four study sites representing a cross section of malaria transmission risk and housing style. Structured questionnaires, DL visual inspections and group interviews (GIs) were completed with household heads at 12- and 36-months post-installation. Three DL samples were collected from all households in which it remained 36-months post-installation to evaluate the bio-efficacy of DL on *Anopheles* mosquitoes. Bio-efficacy testing followed WHO guidelines for the evaluation of indoor residual spraying.

**Results:**

The DL was still intact in 86 and 39% of study homes at the two time periods, respectively. In homes in which the DL was still intact, 92% of household heads considered the appearance at 12-months post installation to be the same as, or better than, that at installation compared to 59% at 36-months post-installation. GIs at both time points confirmed continuing high acceptance of DL, based in large part of the perceived attractiveness and functionality of the material. However, participants frequently asserted that they, or their family members, had ceased or reduced their use of mosquito nets as a result of the DL installation. A total of 16 houses were sampled for bio-efficacy testing across the 4 study sites at 36-months post-installation. Overall, combining all sites and samples, both knock-down at 30 min and mortality at 24 h were 100%.

**Conclusions:**

The ZeroVector^®^ DL installation remained highly acceptable at 36-months post-installation, the material and fixtures proved durable and the efficacy against malaria vectors did not decrease. However, the DL material had been removed from over 50% of the original study homes 3 years post-installation, largely due to deteriorating housing infrastructure. Furthermore, the presence of the DL installation appeared to reduce ITN use among many participating householders. The study findings suggest DL may not be an appropriate vector control method for large-scale use in the contemporary PNG malaria control programme.

## Background

Durable lining (DL) is an insecticide-impregnated polyethylene sheeting designed to cover the interior walls of domestic dwellings as a form of vector control. Entomological studies have demonstrated the effectiveness of DL impregnated with various forms of insecticide under a range of field conditions [1–4], reporting vector mortality as high as 100% 1 year post-installation [4]. DL has been successfully installed across a range of housing types and geographic settings with consistently high user-acceptance [4–8]. Furthermore, DL has been identified as a preferred method of vector control, even in comparison to insecticide-treated mosquito nets (ITN) or indoor residual spraying (IRS), among field trial participants [5–7].

The optimal role of DL in vector control programing remains uncertain despite its apparent efficacy, feasibility and acceptability. DL has been proposed as a complementary intervention to be used alongside ITNs [1, 9, 10]. However, DL confers less personal protection in comparison to ITNs and studies have shown no additional protective benefit from combining DL with ITNs when both share the same class of insecticide [9]. The use of two vector control interventions employing the same insecticide, especially if used on scale, may also accelerate the development of insecticide resistance [11]. As such, a DL/ITN combination may best be limited to settings where ITN effectiveness is waning due to developing pyrethroid resistance and where non-pyrethroid impregnated DL is available. Experimental evidence suggests a DL/ITN combination under these circumstances can restore vector mortality and personal protection measures to levels comparable with ITN use in a context of low insecticide resistance [1, 10, 12], although a DL/ITN combination may still be ineffective in settings with multiple insecticide resistant vector populations [13].

Durable lining is perhaps more commonly considered an alternative to IRS in vector control programming [1, 3, 7]. Pyrethroid-impregnated DL can remain active for a 3- to 5 year period unlike IRS which requires reapplication annually or biannually [3]. Pyrethroid-impregnated DL has also been proven more efficacious than IRS 12-months post-application [6]. Thus, DL clearly presents as a potentially longer lasting, less labour-intensive and more efficacious vector control method as compared to IRS. Nevertheless, questions remain as to whether DL is a better alternative to IRS in all contexts or only in high-transmission settings or settings where surveillance systems and public health infrastructure are poorly developed [1, 3].

The ongoing debate as to the optimal role of DL in vector control programing is hampered in part by a paucity of longer-term investigation. To the authors’ knowledge, no published studies have examined the acceptability, durability or bio-efficacy of DL beyond 12- to 15-months post-installation. Longer-term investigation is essential as a central premise of the IRS-DL comparison is that the latter will remain in effective use for a 3-to-5 year period. The potential for long-term bio-efficacy of pyrethroid-impregnated materials has been well established in an ITN context [14], although not yet for DL. The effective use of DL over the longer-term is also not merely a question of potential insecticide duration. Living conditions within the household may undermine or accelerate loss of insecticidal activity, the DL installation and material must remain intact and the householders must choose to maintain the installation. Any one of these factors may reduce the duration of DL effectiveness, negating the potential advantages of DL over IRS. Furthermore, the impact of a DL installation on householder’s use of complementary forms of vector control is not well understood. An earlier study from Papua New Guinea suggested a DL installation may reduce ITN use at the household level, potentially increasing the risk of malaria transmission [5]. However, this study only examined the impact of DL on ITN use in the 4-week period immediately following installation and householder preferences and/or practices may have changed thereafter.

To address the paucity of longer-term investigation, this study examines the acceptability, durability and bio-efficacy of pyrethroid-impregnated DL over a 3 year period post-installation. The study was based in Papua New Guinea (PNG), a malaria-endemic country in the Western Pacific, and is a follow-up to a previously published feasibility and initial acceptability study [5].

## Methods

### Study sites

This study was conducted in four sites across PNG in which ZeroVector^®^ DL had previously been installed as part of a feasibility and initial acceptability study [5]. The sites included Lokwitua (New Ireland province, islands village), Nauna (Madang province, lowlands village), Masumave (Eastern Highlands province, highlands village) and Kofi Roots (Eastern Highlands province, peri-urban). A full description of the study sites, the housing types and the DL installation process are presented in this earlier publication. To briefly summarize, DL was installed in forty homes across four sites (10 homes per site) purposively selected on the basis that they represented a cross section of malaria transmission risk and housing style. Overall, 52.5% of the homes were constructed of traditional materials (e.g. bamboo, rough cut ‘untreated’ timber), 25% were constructed of commercially available materials (e.g. plywood, iron sheeting) and 22.5% were a mix of the two. The DL was installed by a field team trained in standard ZeroVector^®^ DL installation protocols. The locations of all participating homes were geo-referenced.

### Procedure

A researcher involved in the original DL installation in 2012 revisited each site at both 12- and 36-months post-installation. Using geo reference data and local contacts, the homes in which DL had been originally installed were located. An interviewer administered questionnaire was then completed with the household head alongside a visual inspection of the DL, guided by a structured checklist. Wherever possible, household items obscuring the DL material were moved to allow a full visual inspection of the DL installation and integrity. Items on the questionnaire included: perceived effectiveness of DL, DL cleaning and maintenance, experience of illness, perceived attractiveness of DL and the use of complementary vector control/personal protection measures. The visual inspection centred on the general appearance of the DL, potential modifications to the original installation, the integrity of the DL (e.g. presence of holes, abrasions or scorch marks) and the integrity of the DL fixtures (e.g. were the original fixtures intact). An ‘abrasion’ was defined as any fraying of the DL weave and each observed abrasion was measured on a four-point visual scale from 1 (minor) to 4 (severe).

At each study site, at both 12- and 36-months post installation, one adult male and/or female member from each household in which the DL remained intact was invited to participate in a group interview (GI). The criteria for inclusion in the GIs were: that he/she must normally reside in a housing structure in which the DL was installed; that he/she must be the male/female household head or have been authorized by the household head to speak on his/her behalf; and that the household representative must be 18 years of age or older. All GIs followed a schedule variously focusing on initial and subsequent impressions of DL, installation and ‘user’ experiences, and the expected and realized advantages and disadvantages of DL, including real or perceived side effects.

During the 36-months post installation survey, DL samples were sought from all households in which the material remained affixed to evaluate the residual action or bio-efficacy of DL on *Anopheles* mosquitoes after 3 years of normal use in the field. Upon verbal approval by the household head, three 25 × 25 cm pieces of DL were cut in each selected house at the bottom, middle and top of a wall, then replaced by new DL pieces. Samples were folded and stored individually within aluminium foil and kept refrigerated until transported to the laboratory. The bio-efficacy testing followed WHO guidelines for the evaluation of indoor residual spraying [15] and was done using *Anopheles farauti* sensu stricto female mosquitoes aged 2–5 days reared in the insectary. Four plastic conical chambers from WHO bio-assay kits were pinned on each DL piece, then 10 mosquitoes were introduced in each chamber and exposed to the insecticide-treated material for 30 min (40 mosquitoes per DL sample). Knocked-down mosquitoes were counted at 10, 20 and 30 min, then placed in clean cups with 10% sugar solution provided and kept in a shaded place at 28–30 °C and 70–90% relative humidity. Dead mosquitoes were counted after a recovery period of 24 h. An untreated bed net was used as negative control, and a piece of new DL was used as positive control.

### Data analysis

All quantitative data was entered onto an Excel spreadsheet. Descriptive statistics were conducted as required. All interviews were recorded on a digital voice recorder, transcribed verbatim, translated into English, and entered into NVIVO 9. A thematic analysis of the GI data was conducted as informed by a general inductive methodology [16]. GI data were independently coded by two investigators. The two coders compared and agreed upon codes and emerging themes after independent coding, resolving disagreement by consensus opinion or by the creation of new, mutually agreeable, codes. The two indicators of DL bio-efficacy were the knock-down rate of mosquitoes after 30 min exposure and the mortality rate after 24 h recovery period. Data were analysed per site and sample position on the wall (i.e. top, middle or bottom). Low mortality in negative controls (Less than 5%) did not require calculation of corrected mortality rates.

## Results

### Sample

The household questionnaire and DL visual inspection was completed in 37 out of the original 40 study homes at 12-months post-installation and 38/40 homes at 36-months post-installation. Table 1 identifies the number of questionnaires/visual inspections completed at each time point by site. A total of 8 GIs were conducted across the two time periods, one at each site at both 12- and 36-months post-installation. A total of 25 and 20 individuals participated across the GIs at each time point, respectively. A DL sample for bio-efficacy testing was obtained from 16/17 homes in which the DL remained intact or modified at 36-months post-installation.

**Table 1:** Durability of insecticide treated plastic sheeting 12- and 36-months post installation

Site	Time (months)	HH no	Installation status no (%)			DL integrity no (%)^a^			Fixture integrity no (%)^a^		
			Intact	Modified	Removed	Holes	Abrasions	Scorched	Intact	Loose^b^	Failed^c^
Islands village	12	10	10 (100)	0	0	6 (60)	7 (70)	0	5 (50)	4 (40)	1 (10)
	36	10	4 (40)	0	6 (60)	2 (5)	2 (50)	1 (25)	2 (50)	2 (50)	0
Lowlands village	12	10	8 (80)	1 (10)	1 (10)	4 (45)	6 (67)	0	4 (45)	5 (55)	0
	36	9	3 (33)	1 (11)	5 (56)	4 (100)	3 (75)	0	2 (50)	2 (50)	0
Highlands village	12	8	7 (88)	1 (12)	0	6 (75)	5 (63)	1 (13)	4 (50)	4 (50)	0
	36	9	5 (56)	1 (11)	3 (33)	6 (100)	3 (50)	5 (83)	3 (50)	3 (50)	0
Highlands urban	12	9	7 (78)	1 (11)	1(11)	2 (25)	4 (50)	0	6 (75)	2 (25)	0
	36	10	3 (30)	0	7 (70)	1 (33)	1 (33)	0	2 (67)	1 (33)	0
Overall	12	37	32 (86)	3 (8)	2 (5)	18 (51)	22 (63)	1 (3)	19 (54)	15 (43)	1 (3)
	36	38	15 (39)	2 (6)	21 (55)	13 (76)	9 (53)	6 (35)	9 (53)	8 (47)	0

^a^Excludes households in which DL was removed

^b^Defined as loose or missing fixtures (nails) or slackness in the DL to the degree that the material may be pulled away from the wall by a distance of at least 50 cm

^c^Defined as DL no longer affixed to one or more parts of the interior wall due to a failed fixture (as opposed to deliberate removal)

### Questionnaire/visual inspection

Table 1 depicts the installation status of DL in the study homes at each time point as well as the integrity of the DL and the fixtures (primarily nails) used to hold it in place. As shown, at 12-months post installation the DL was still intact (i.e. no modification to the original installation) in 86% of study homes, modified in 8% and removed from 5%. At 36-months post-installation the DL was still intact in 39% of study homes, modified in 6% and removed from 55%. Reported modifications across the two time periods included cutting out a portion of the DL in order to store items against the interior wall (n = 2), because it had been damaged by children (n = 1) or fire (n = 1) or because it had been rehung in a new house (n = 1).

In terms of housing type, the DL had been removed from 50% (10/20) of the observed homes built from traditional materials at 36-months post-installation, 50% (4/8) of observed homes built from a mix of traditional and commercial materials and 70% (3/10) of observed homes built from commercial materials. Reported reasons for removing the DL by housing type included: traditional—destruction of the home due to wear and tear or family conflict (n = 8), children spoiling the DL installation (n = 1) and heat discomfort (n = 1); mixed materials—destruction of the home due to wear and tear or elements (n = 4); and commercial—construction of new home (n = 1), children spoiling the DL installation (n = 2), DL no longer considered effective (n = 2), DL no longer considered attractive (n = 1) and reasons unknown (n = 1). Of the installations still intact or modified, holes were observed in the DL in 51% (18/35) of homes 12-months post-installation and 76% (13/17) at 36-months post-installation. In homes with at least one hole in the DL, the median number of holes observed was 2 at both 12- and 36-months post-installation (range 1–6 and 1–8, respectively). Median hole size (height × width) was 72 and 90 cm at 12- and 36-months post-installation, respectively (range 8.75–1386 cm and 4–483 cm, respectively). Abrasions were observed in 63% (22/35) of homes with the DL intact or modified at 12-months post-installation and 53% (9/17) at 36-months. The median abrasion rating was 1 at both 12- and 36-months post installation (range 1–4 & 1–3, respectively). A scorch mark was observed on the DL in only one home at 12-months post-installation and six homes at 36-months. All scorch marks were caused by an internal open cooking fire (n = 5) or a candle (n = 2).

The fixtures were fully intact and the DL tight against the interior wall in 54% (19/35) of study homes in which the DL remained intact or modified at 12-months post-installation and 53% (9/17) at 36-months. The DL fixture was loose in 43% (15/35) of homes in which it remained installed at 12-months post-installation and 47% (8/17) at 36-months. The median number of loose ‘areas’ was 1 at both time-points (range 1–5 and 1–3, respectively). The average ‘amount’ of slack in each loose area (measured by pulling the material tight in a horizontal direction and then measuring the distance between the wall and the DL in the loose area) was 42.7 and 26.6 cm, respectively. The fixture had failed in part in one home at 12-months post-installation to the extent that the DL was hanging loose exposing the interior wall. The DL installation had been removed from this home at 36-months follow-up.

Table 2 depicts the perceived appearance of the DL, and its perceived effectiveness, as compared to the time of installation. As shown, 89% (31/35) of household heads in homes in which the DL was still intact considered the appearance at 12-months post installation to be the same as that at installation, 9% thought the appearance had deteriorated and 3% thought it had improved. At 36-months post-installation the comparable percentages were 59, 41 and 0%, respectively. 57% of household heads considered the DL to be less effective at 12-month follow-up as compared to installation, 37% considered the effectiveness to be about the same and 6% considered the DL to have become more effective. At 36-months post-installation the comparable percentages were 76, 24 and 0%, respectively. One participant (Highlands urban) at the 12-month follow-up reported one or more household members having suffered a malaria infection since DL installation, although in this case the affected individuals slept in a room without DL coverage. No participant reported a household member suffering a malaria infection in the period between 12- and 36-months follow-up.

**Table 2:** Perceived appearance and perceived effectiveness of DL at 12- and 36-months follow-up as compared to the time of installation

Site	Time (months)	HH no^a^	Perceived appearance no (%)			Perceived effectiveness no (%)		
			Better	Same	Worse	Better	Same	Worse
Islands village	12	10	0	9 (90)	1 (10)	0	2 (20)	8 (80)
	36	4	0	1 (25)	3 (75)	0	1 (25)	3 (75)
Lowlands village	12	9	0	8 (89)	1 (11)	0	3 (33)	6 (67)
	36	4	0	2 (50)	2 (50)	0	2 (50)	2 (50)
Highlands village	12	8	1 (13)	7 (87)	0	2 (25)	3 (36)	3 (36)
	36	6	0	4 (67)	2 (33)	0	0	6 (100)
Highlands urban	12	8	0	7 (87)	1 (1)	0	5 (64)	3 (36)
	36	3	0	3 (100)	0	0	1 (33)	2 (67)
Overall	12	35	1 (3)	31 (89)	3 (9)	2 (6)	13 (37)	20 (57)
	36	17	0	10 (59)	7 (41)	0	4 (24)	13 (76)

^a^Only includes home with a full or partially intact DL installation

At 12 months post installation, 91% (32/35) of participants from a home in which the DL remained intact or modified reported that at least one ITN was available for use within the household (median 2, range 1–5). A total of 139 individuals were reported living in these 32 homes, 42% (59/139) of whom had reportedly not slept under a mosquito net the night prior to survey. At 36 months post installation, 76.5% (13/17) of participants from a home in which the DL remained intact or modified reported that at least one ITN was available for use within the household (median 2, range 1–4). A total of 34 individuals were reported living in these 17 homes, 64.7% (22/34) of whom had reportedly not slept under a mosquito net the night prior to survey.

### Group interviews

A majority of focus group participants still considered the DL to be effective at 12-months post installation, although there was some suggestion of decreasing effectiveness from the lowland and islands sites. However, by 36-months post-installation most participants from all sites were reporting a perceived decrease in the effectiveness of DL. Interestingly, it was widely reported at this time that the decrease in effectiveness was evident within months post-installation:

*“I suppose it [the insecticide in the DL] has lasted about four months. After four [months] and beyond, everything diminished. It has no more power, it is powerless.” (Lowlands village, 36*-*months, participant 2).*

Despite the reduction in perceived effectiveness, no participant at either time point indicated that they planned to remove the material from their home and most expressed interest in obtaining new DL to replace the existing installation or for use in a new home:

“*The net is making the house look nice so I will leave it. And if you give us new ones [DL] then I shall remove it and put up new one or such”. (Highlands urban, 36*-*months, participant 3).*

Several factors influenced the continued acceptability of the DL material among focus group participants. The perceived attractiveness of the material was an important consideration in this regard with most focus group participants at both 12- and 36-months post-installation reporting the DL improved the appearance of their home. This is not to suggest that the degree of perceived attractiveness remained unchanged, at each time-point an increasing number of participants noted the overall appearance of the DL had diminished somewhat, yet even in a diminished state the interior household was still considered enhanced as a result of the wall lining:

*“Yes, initially it [the DL] looked very nice. It made the house look nice, but now that it is losing its colours or maybe the dust covered it so its colours are fading. But it’s still looking nice on the wall as it is.” (Highlands urban, 36*-*months, participant 4).*

Nevertheless, not one participant suggested that they would install the DL for appearances sake alone. Thus, perceived function was essential to the initial and continued acceptability of DL and several ‘functions’ were described. The first, and most important, was its intended function as a form of malaria control. Participants at both time points frequently recounted a sudden and dramatic reduction in household insects and pests immediately after installation. While this perceived level of impact was not thought to have been sustained at 36-months post-installation, the DL was generally considered to have retained some degree of insecticidal activity and malaria episodes at the household level were not reported:

“*For myself, when this thing [DL] was there I see that me or my family members had never been sick with malaria since this thing was installed. Not one of us was infected with malaria. That is why I like that thing.” (Islands village, 36*-*months, participant 1).*

Householders from the cooler Highlands region repeatedly suggested that the material warmed the house which was also considered a desirable function of the DL. A ‘warming’ benefit was not reported by householders from the hotter lowlands sites, but neither did they report household warming as a negative (i.e. rendering the house too hot for comfort). In addition, participants from all sites, and across both time-points, expressed a preference for the DL over other forms of malaria control (including mosquito nets) due to its comparative ease of use. This, too, may be considered an appealing functionality-based feature of the DL.

*“Previously we used to do the work of tying up nets and sleep and even in the night to wake up and tie up nets and now this green net is here, sorry blue net [DL], that we do not have the hard work of tying the nets. It’s [DL] on the wall helping us to kill mosquitoes so we just sleep relaxing”(Highlands village, 36*-*months, participant 2).*

Further contributing to the continued appeal of the DL was the lack of reported side-effects experienced by focus group participants. No participant reported a serious side-effect or problem associated with the DL at either time point and any references to side-effects were generally limited to mild skin irritation following contact with the material in the days immediately following installation. Indeed, for some participants the DL was appealing because it did not incur the same side-effects as experienced when using ITN:

*“I don’t like using the mosquito net. Sometimes I have shortness of breath”. (Islands village, 12*-*months, participant 2).*

Appraisal of the DL at 12- and 36-month follow-up was primarily, but not exclusively, positive. A number of participants suggested the DL material was not sufficiently durable and was prone to damage, although this was not a consensus opinion. The most frequently reported cause of damage were children playing with or near the wall lining. A few participants expressed a preference for an alternative colour, although no specific colour was consistently requested. A number of participants at the 12-month follow-up stated that the material emitted an unpleasant odour in the weeks after installation, but was not a problem thereafter. No comments on odour were made at the 36-month follow-up. No participant reported any attempt to repair damage to any portion of the DL at either time point. The lack of repair, despite damage, was often attributed to a lack of knowledge as to how to carry out a repair or a perception of the DL as a disposable material:

*“When it [DL] is in the house, it is just there, but when the house gets old and when we dismantle it, or like when the roof of the house has a hole or when the rain leaks in and damages it, it is like waste, we just remove it and throw it away.” (Lowlands village, 36*-*months, participant 3).*

The most concerning theme to emerge from the focus group discussions was the frequently asserted contention, at both time points, that participants and their families had ceased or reduced their use of mosquito nets as a result of the DL installation. This was always expressed in a positive manner and typically framed as a benefit of the DL relative to ITN (as illustrated by a previous quote). Not all participants reported ITN use prior to DL installation and a number of participants persisted with ITN use after DL installation; however, the focus group comment was highly suggestive of reduced ITN use overall across the participating households across all sites and there was no comment to suggest any awareness that this resulting reduction in ITN use increased risk of malaria infection. Rather, participants widely expressed the view that DL offered a superior level of personal protection:

*“The nets [ITN provided during a national mass distribution campaign] that were brought to us were treated, but it only takes a week for the treatment to finish. Also the mosquitoes tend to fly around the net and buzz around, but with the durable lining [DL] we have seen the insects fall on the ground and die. We want the ones [DL] that kill the insects and not the ones [ITN] that don’t kill the insects because cockroaches, mosquitoes and rats cause sicknesses. When we leave left over food out in the open, after rats have eaten, we can get sick because it has germs. When cockroaches sit on our food, we can get sick. This net [ITN] in the house doesn’t seem to serve its purpose, but this net [DL] you people brought, honestly it works.” (Highlands village, 12*-*months, participant 3).*

*“[DL] is better than the mosquito net and the other thing is that I can breathe properly when I’m sleeping, but in the mosquito net I feel that I am breathing in all the medicine/treatment from the net. Now that we are using this [the DL], we don’t want to use the mosquito net, our nets are piling up there. I am ready to sell mine. We don’t really like mosquito nets. These nets [DL] are better than mosquito nets. For me and my families good I’m saying this.” (Islands village, 12*-*months, participant 5).*

### Bio-efficacy testing

A total of 16 houses were sampled for DL pieces across the 4 study sites. Overall, combining all sites and positions, both proportions of mosquitoes knocked-down at 30 min and dead at 24 h were 100% (Table 3). Optimal knock-down and killing efficacy were observed for each study site, as well as each position on the wall (bottom, middle and top). Based on those two indicators, the results were identical to the control tests using new DL.

**Table 3:** Proportion of *Anopheles farauti* s.s. mosquitoes knocked-down (KD) at 30 min and dead at 24 h, by study site and height of DL samples

Site	DL sample position											
	Top			Middle			Bottom			All Positions		
	n	KD (%)	Dead (%)	n	KD (%)	Dead (%)	n	KD (%)	Dead (%)	n	KD (%)	Dead (%)
Islands village (n = 4)	160	100	100	160	100	100	160	100	100	480	100	100
Lowlands village (n = 4)	163	100	100	161	100	100	160	100	100	484	100	100
Highlands village (n = 5)	165	100	100	209	100	100	219	100	100	593	100	100
Highlands urban (n = 3)	120	100	100	122	100	100	121	100	100	363	100	100
Overall	608	100	100	652	100	100	660	100	100	1920	100	100

*n* number of mosquitoes exposed, *KD* knock-down after the 30 min exposure, *Dead* mortality after the 24 h recovery period

## Discussion

An earlier study reported high user-acceptability of DL in four distinct settings in PNG immediately following installation [5] and the findings presented in this paper strongly suggest user-acceptability remains high 3 years later. A majority of household heads in the homes in which DL remained installed at both 12- and 36-months post installation still considered the installation to be attractive and functional, even if perceived to be somewhat less attractive and less effective than when first installed, and there was widespread interest in obtaining new DL material. In the 21 homes in which the DL had been removed by 36-months post-installation, there were only four cases where the removal was attributed to a decline in product acceptability. These included a perceived loss of effectiveness (n = 2), heat discomfort and a perceived loss of attractiveness.

The study findings were largely positive with respect to the durability of both the DL fixture and the DL material. In those homes in which the DL installation remained, holes, abrasions and/or scorch marks were observed on at least one part of the product at both 12- and 36-months post installation (with an increasing percentage over time). However, holes were limited to a median of two per household at both time points, abrasions were typically rated ‘minor’ and scorch marks were evident in only six homes. Fixtures remained fully intact in approximately 50% of these households at both time points. In those homes with a loose fixture, the affected area was typically small and there was only one observed case of a failed fixture (defined as the fixture failing to the point where the interior wall was exposed). Thus, although some deterioration of both the DL product and fixture was often evident, the level of deterioration was relatively minimal and the product and fixture remained largely in sound condition in those homes in which the installation remained. GI participants frequently identified “children” and their handling of the product as a threat to DL longevity, although the observational data only revealed a few instances of severe damage or installation failure as a result of child-related damage.

Despite the apparent durability of the DL product and fixture, by 36-months post installation the DL had been removed from over half of the original study homes and in most of these cases the product had been removed due to deterioration of the housing infrastructure to which the product had been affixed. In other words, while the ZeroVector^®^ material proved relatively durable, the material used in the construction of traditional PNG housing types (typically untreated, locally sourced wood and bush material) was not. These findings suggest that if DL were to be implemented on a larger scale in PNG, its effective duration would be largely determined by the age and condition of the respective housing material at the time of installation rather than the product itself. This is an important consideration as approximately 60% of homes in PNG are constructed out of traditional ‘bush’ materials [17]. It was of note that not one householder reported removing the DL from the deteriorating infrastructure and re-installing in the newly built home (or new interior wall on the existing home). The nail-based fixtures may have prevented this as they were not easily removed without appropriate tools (which may not be commonly available at the rural household level in PNG) and, therefore, may not be the best fixture for use in PNG homes. This contrasts with findings from a recent study in Ghana where nail-based fixtures were considered ideal for DL installation in homes constructed out of clay or concrete rendered mud walls [18].

The study findings further suggest that DL installations are also likely to be removed from PNG homes built from more durable ‘commercial’ building products such as plywood, brick or iron within 3 years of installation. Study participants from these more durable homes reported a wider variety of reasons for removing the DL material from their walls, although a common element was a perceived reduction in attractiveness or effectiveness suggestive of a lower ‘acceptance’ threshold among residents of housing types constructed out of commercial products. Thus, housing type would not appear to greatly influence the effective lifespan of DL in the PNG context.

In those homes in which DL remained intact, 57% of household heads perceived the effectiveness of the product to have declined at 12-months post-installation increasing to 76% at 36-months post-installation. This finding was echoed in the group interviews where participants widely reported a perceived increase in mosquito and insect numbers in the household following the rapid reduction in the period immediately following installation. The bio-efficacy testing did not support these perceptions of decreasing insecticidal potency, suggesting participant perceptions of reduced product effectiveness were not synonymous with ineffectiveness.

To the authors’ knowledge, this is the first report of the bio-efficacy of durable lining against malaria vectors after several years of field use. The results are based on a limited number of households, but showed clearly an extremely good residual activity of ZeroVector^®^ DL in rural Papua New Guinea, with no detectable loss of killing efficacy following WHO guidelines for such tests. In terms of insecticidal effectiveness, DL would therefore appear to be a potentially better alternative to indoor residual spraying, with the major advantage of remaining efficient for a minimum of 3 years after installation, while indoor spraying can have a residual activity for 6 months at best. The results were gathered in the laboratory and involved experimental exposure to DL samples in cone bio-assays that may not fully reflect how wild indoor resting mosquitoes are impacted in operational conditions. Thus, additional studies would be needed to assess the actual benefits and limitations of DL from a transmission and entomological perspective in the context of Papua New Guinea. The colony mosquitoes used belong to one of the major malaria vector species in the country, *An. farauti* s.s., found to be fully susceptible to pyrethroid insecticides [19]. While these mosquitoes were readily knocked-down or killed by pyrethroid-treated DL in the laboratory, they also exhibit a tendency to feed and rest outdoors which might limit the probability of contact with the treated material, and potentially compromise the effectiveness of DL to reduce malaria transmission.

This study was designed in large part to inform debate pertaining to the best use of DL in vector control programmes. Findings relevant to both DL/ITN combinations and DL as an alternative to IRS are evident. To date, the potential use of a DL/ITN combination has been primarily considered in terms of the potential for greater vector mortality that may arise from increasing the volume of insecticide-treated surfaces within a home; a hypothesis largely examined in experimental contexts [1, 9, 12]. However, this study and its predecessor [5] highlight an important behavioural consideration that may undermine the potential effectiveness of a DL/ITN combination applied in a real world setting; namely, that many householders may choose to abandon the use of the more intrusive and intensive ITNs if provided with a DL installation.

Whilst experimental trials have demonstrated a potential benefit from utilising a DL/ITN combination [1, 10, 12], the practical application of any such combination could paradoxically result in greater vector borne disease infection if householders subsequently choose not to use their ITNs as DL confers relatively minimal personal protection against mosquito bites [3, 9]. Firm conclusions cannot be drawn from this study as ITN use was not examined among non-study households in the participating villages. Nevertheless, the DL trials took place at a time when ITN ownership and usage were increasing across PNG [20, 21] and the ITN usage rate among householders with DL intact at 36 months follow-up (35.3%) was substantially lower than the national average of 53.9% recorded at a similar time period [22]. A recent study has suggested partial DL installations remain as effective as full wall surface installations in the control of sand flies [23]. It may therefore be worth examining in future investigations the impact of DL installation size (measured in terms of the extent of wall surface covered) on householder’s ITN use (hypothesising that a ‘smaller’ DL installation will have a lesser impact). ITN longevity has also not been investigated in PNG, so it remains uncertain as to how the effective life span of DL compares to that of an ITN in this context. The reported increase in household ITN ownership at the national level following 3-year ITN distribution cycles suggests a lifespan of greater than 3 years [22], although more detailed investigation is required.

The study findings further suggest that DL may not be a vastly superior alternative to IRS in a PNG context. A key potential advantage of pyrethroid-impregnated DL over IRS is that, due to its long-lasting insecticidal treatment, it may remain in effective use for up to a 5 year period following installation. As the original DL installation remained intact in fewer than fifty percent of homes at the 36-month follow-up, then this study suggests the actual effective use duration of DL will be considerably briefer in PNG than that which may be realized elsewhere. As discussed above, the major limiting factor is the poor quality, and relatively brief lifespan, of traditional PNG home building materials. This limitation would remain irrespective of the type of insecticide used in a DL product. DL use may still be appropriate if the cost of installation is similar to the cost of an IRS campaign (given the relatively brief effective duration of IRS), although previous studies have articulated concerns about the cost and complexity of DL installation as compared to IRS application [5, 24].

Until such time as reliable cost-effectiveness studies have been completed, then on the basis of the reported findings, DL may be better considered as a niche tool for labour camps or in emergency situations in the PNG setting as opposed to an alternative to IRS. This finding also raises the possibility that insecticide-treated wall netting may be a better option for widespread use in PNG if an alternative to IRS is still sought. Net hangings are simpler to install than DL, are likely to be easier to remove from deteriorating housing infrastructure and rehung in new homes and have proven efficacy as a vector control tool [24]. Product feasibility and acceptability trials would still need to be conducted in a PNG setting before firm conclusions could be drawn as to the potential of net hangings as an alternative to IRS. Similarly, the potential impact on ITN use would also need to be examined.

This study was not without limitation. The number of homes included in the trial was relatively small and, as such, study findings pertaining to the potential impact of a DL installation on ITN use and the user experience of DL should not be broadly generalized without further investigation. The study homes were also not representative of all housing types in PNG. Nevertheless, the study sites were purposely selected to represent a range of housing types and the housing materials used across the participating homes were broadly representative of materials used throughout PNG, even if the housing structures may vary. Bio-efficacy testing was not conducted at the time of DL installation or at 12-months follow-up, so it was not possible to measure the degree and rate of degradation in insecticide activity across time.

## Conclusion

The ZeroVector^®^ DL installation remained highly acceptable at 36-months post-installation, the material and fixtures proved durable and the efficacy against malaria vectors did not decrease. However, the DL material had been removed from over 50% of the original study homes 3 years post-installation, largely due to deteriorating housing infrastructure. Furthermore, the presence of the DL installation appeared to reduce ITN use among many participating householders. The study findings suggest DL may not be an appropriate vector control method for large scale use in the contemporary PNG malaria control programme in combination with ITNs and cost-effectiveness studies would be needed to determine if DL is a viable alternative to IRS whether on a large or even smaller scale.

## Abbreviations

DL: durable lining
GI: group interview
IRS: indoor residual spraying
ITN: insecticide-treated net
PNG: Papua New Guinea
WHO: World Health Organization
